# TLR4 and SARM1 modulate survival and chemoresistance in an HPV-positive cervical cancer cell line

**DOI:** 10.1038/s41598-022-09980-6

**Published:** 2022-04-25

**Authors:** Mirian Galliote Morale, Rodrigo Esaki Tamura, Ricardo Cintra, Natália Meneses Araújo, Luisa Lina Villa

**Affiliations:** 1 Departamento de Ciências Biológicas, Universidade Federal de São Paulo (UNIFESP), Diadema, SP Brazil; 2 Department of Biochemistry, Instituto de Química, Universidade de São Paulo, São Paulo, SP Brazil; 3 Centro de Investigação Translacional em Oncologia, Instituto do Câncer do Estado de São Paulo (ICESP), São Paulo, Brazil; 4 Department of Radiology and Oncology, Faculdade de Medicina, Universidade de São Paulo, São Paulo, Brazil

**Keywords:** Cervical cancer, Pattern recognition receptors

## Abstract

Human Papillomavirus is responsible for a wide range of mucosal lesions and tumors. The immune system participate in tumorigenesis in different ways. For example, signaling pathways triggered by Toll-like receptors (TLR) play a role in chemotherapy resistance in several tumor types and are candidates for contributing to the development of HPV-induced tumors. Here, we studied the receptor TLR4 and the adaptor molecule SARM1 in HeLa cells, an HPV-positive cervical cancer cell line. Knocking out of these genes individually proved to be important for maintaining cell viability and proliferation. TLR4 knock out cells were more sensitive to cisplatin treatment, which was illustrated by an increased frequency of apoptotic cells. Furthermore, TLR4 and SARM1 modulated ROS production, which was induced by cell death in response to cisplatin. In conclusion, TLR4 and SARM1 are important for therapy resistance and cervical cancer cell viability and may be relevant clinical targets.

## Introduction

The relationship between Human Papillomavirus (HPV) and cervical cancer is well known and established^1,2^: the key factor for cervical cancer development is a persistent high-risk HPV infection^3^. HPV persistence leads to increased risk of tumor development despite adaptive or innate immunity against the virus^4^. More than 500,000 woman are diagnosed every year with cervical cancer worldwide and half of them will die from the disease^5^.

High-risk HPV E6 and E7 proteins are essential to generate a series of changes in the cell, increasing mutations that will contribute to tumor development^6^. In addition, these viral proteins also deregulate the innate immune system by modifying Toll Like Receptors (TLR) expression, interfering with other antiviral responses, triggering persistence and leading to lesion progression^7^.

TLRs play a pivotal role in innate immunity responses against pathogens and in recognizing cellular stress^8^. Despite being present in normal cells, TLRs can also be expressed and activated in tumors, where they can either unleash antitumor effects or increase tumor progression and metastasis^9–11^. TLR signaling contributes to tumor cell proliferation, cytokines and chemokines release, angiogenesis, cell survival, chemoresistance and hence, tumor progression^12,13^. As such, TLRs are interesting targets for immunotherapy.

The interaction between HPV and TLR has been previously described. Clearance of HPV16 infection is related to increased expression of *TLR2, TLR3, TLR7, TLR8* and *TLR9*^14^. On the other hand, TLR4 has higher expression levels in cervical cancer cells compared to normal cells and its expression levels correlates with increased risk of lymph node metastasis^15,16^. Also, down-regulation of *TLR9* was also observed in HPV16 infected cell lines^17^. Our group reported modulation of NF-kB by high-risk HPV E6 oncoprotein through TLR pathway-related proteins (MyD88, TRAF6, MYD88–adaptor-like (MAL), IRAK2 and IKKε), which potentiated TLR pathway activation^18^.

There are seven described TLR adaptors of which three are classified as regulatory adaptors: SARM (SARM1), BCAP (PIK3AP1) and SCIM P^19^. SARM1 works as a negative TLR regulator, inhibiting the interaction between TRIF adaptor and TLR4 and TLR3^20^. Despite its role in neuronal degeneration, SARM1 has been recently associated with prostate cancer cell line growth and metastasis progression, and has also been found to be overexpressed in HPV-positive tumor cell lines^15,21,22^.

We have previously reported that cervical cancer cell lines expressing E6 and E7 from high-risk HPV types have several alterations on the TLR pathway, such as increased expression of HMGB1, SARM1 and TLR4^15^. Here, we sought to further dissect the function of the TLR pathway in a cervical cancer cell line focusing on the roles of TLR4 and SARM1 on cell survival and cisplatin resistance.

## Results

### Knock out of either TLR4 or SARM1 reduces viability and proliferation of HeLa cells

**Figure 1:**
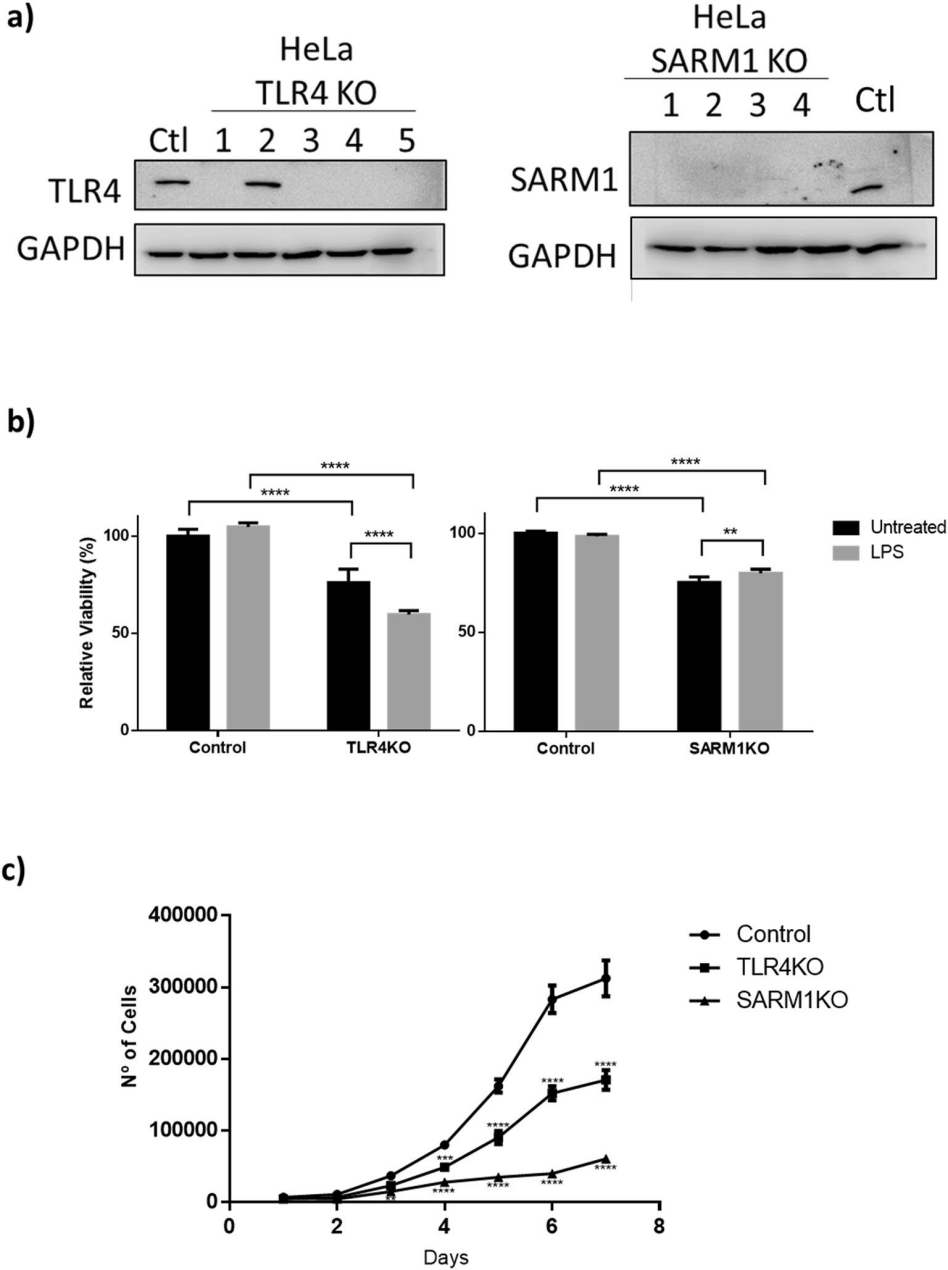
Knockout of TLR4 or SARM1 decreases proliferation and viability of HeLa cells. (**a**) TLR4 and SARM1 protein expression after gene editing. TLR4KO clone 5 and SARM1KO clone 3 were selected for further analysis. (**b**) Cell viability of clones of HeLa cells after TLR4 and SARM1 knockout. Cells were seeded in 96 well-plates, treated with LPS for 48 h and analyzed using PrestoBlue reagent assay. (**c**) Proliferation of TLR4KO, SARM1KO and parental HeLa cells. Cells were seeded in 24-well plates and counted daily in triplicates for 7 days. Data in B and C displayed as mean ± SD. (**p ≤ 0.01; ***p ≤ 0.001; ****p ≤ 0.0001)

To study the role of *TLR4* and *SARM1* in the malignant phenotype of cervical cancer cells, we knocked out these genes individually in HeLa cells using CRISPR/Cas9 and isolated individual clones of the edited cells. Lack of protein expression was confirmed by western blot (Fig. 1a). One clone of each of the *TLR4* and *SARM1* knockout cell lines was then randomly chosen and selected for further analysis. From here on, they will be referred to as TLR4KO (clone 5) and SARM1KO (clone 3).

First, we compared cell viability of TLR4KO and SARM1KO cells to the unedited parental HeLa cells with or without exposure to Lipopolysaccharide (LPS) treatment. Depletion of TLR4 or SARM1 induced approximately 25% reduction of cell viability when compared to the parental HeLa cells (Fig. 1b). Specifically in the case of TLR4KO, loss of viability was further increased by 15% by the LPS treatment (Fig. 1b). In addition, both TLR4KO and SARM1KO cells proliferated less than the parental HeLa cells, with SARM1KO cells showing a reduction of 80% and TLR4KO cells of 45% in proliferation (Fig. 1c).

### Lack of TLR4 and SARM1 reduces the clonogenic and migration potential of HeLa cells

**Figure 2:**
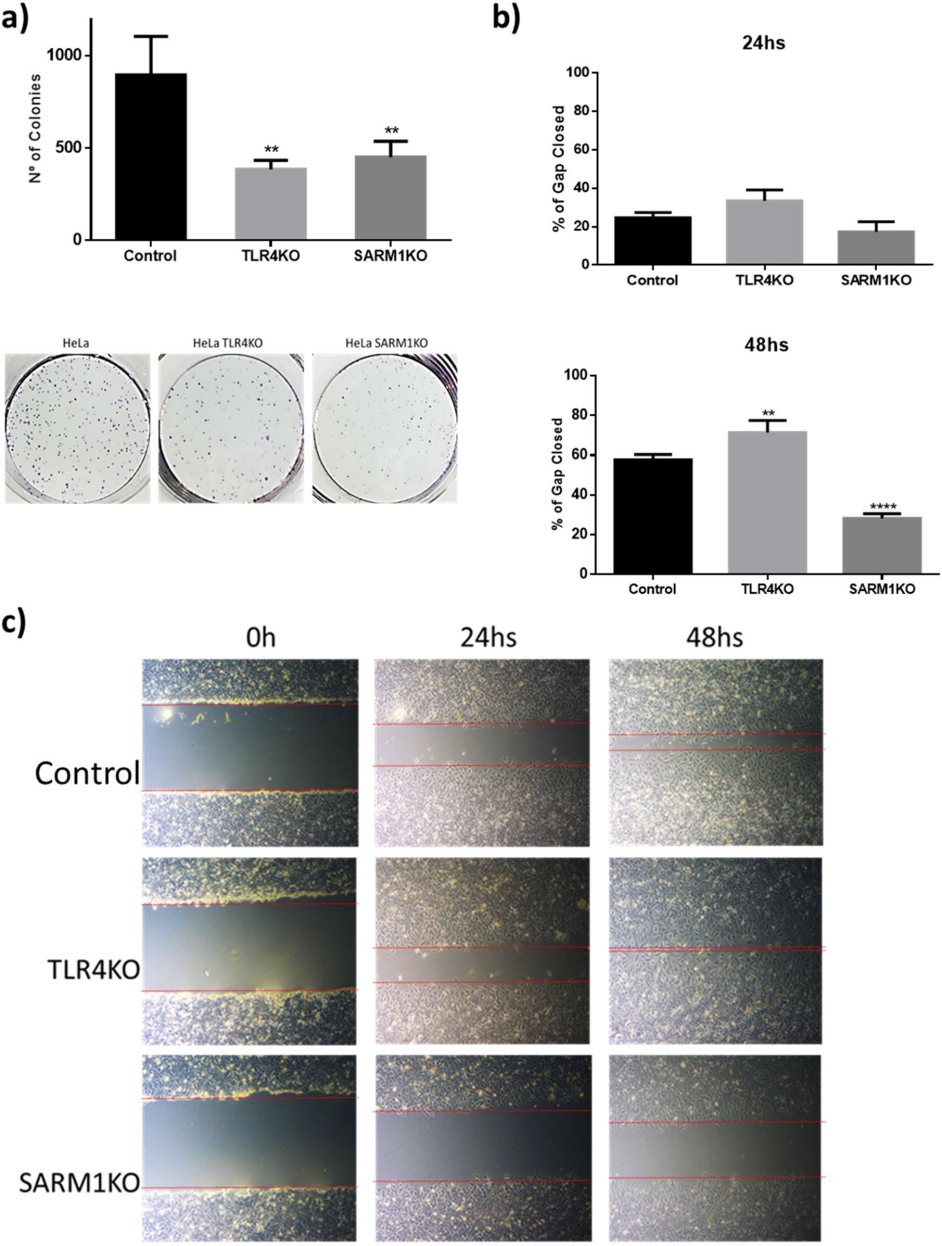
TLR4KO and SARM1KO cells show reduced clonogenic and migration potentials. (**a**) Number of colonies formed by TLR4KO, SARM1KO and parental HeLa cells after 10 days of low-density seeding. 1000 cells of each cell line were seeded in 6-well plates and colony formation was assessed using crystal violet staining. (**b**) Percentage of wound healing by TLR4KO, SARM1KO and parental cells after 24 h and 48 h. (**c**) Representative micrographs of TLR4KO, SARM1KO and parental cells 24 and 48 h after the wound was performed. The wound healing assay was performed after mitomycin C treatment. Data in A and B displayed as mean ± SD. (**p ≤ 0.01; ****p ≤ 0.0001)

We also analyzed the effect of knocking out *TLR4* and *SARM1* on the clonogenic and migratory potential of HeLa cells. We observed that depletion of SARM1 and TLR4 reduced the ability of HeLa cells to form colonies when seeded at low density (Fig. 2a). Furthermore, SARM1KO colonies were significantly smaller, consistent with its pronounced reduction of proliferation (Fig. 1c).

To evaluate the migration potential of the cells, we performed a wound healing test. Cells were previously treated with mitomycin-C to rule out that any observed effect was due to proliferation differences between the parental cells and TLR4KO and SARM1KO cells (Supplementary Fig. S2). Interestingly, SARM1KO cells showed a significant reduction in migration after 48 h (Fig. 2b, c). On the other hand, TLR4KO cells displayed a significant increase in migration compared to parental cells, noticeable after 48 h of the generation of the wound (Fig. 2b, c). Our results confirm that lack of TLR4 and SARM1 interfere with important tumor-related characteristics such as clonogenic and migration potentials.

### TLR4 and SARM1 knockouts sensitize HeLa cells to chemotherapy

**Figure 3:**
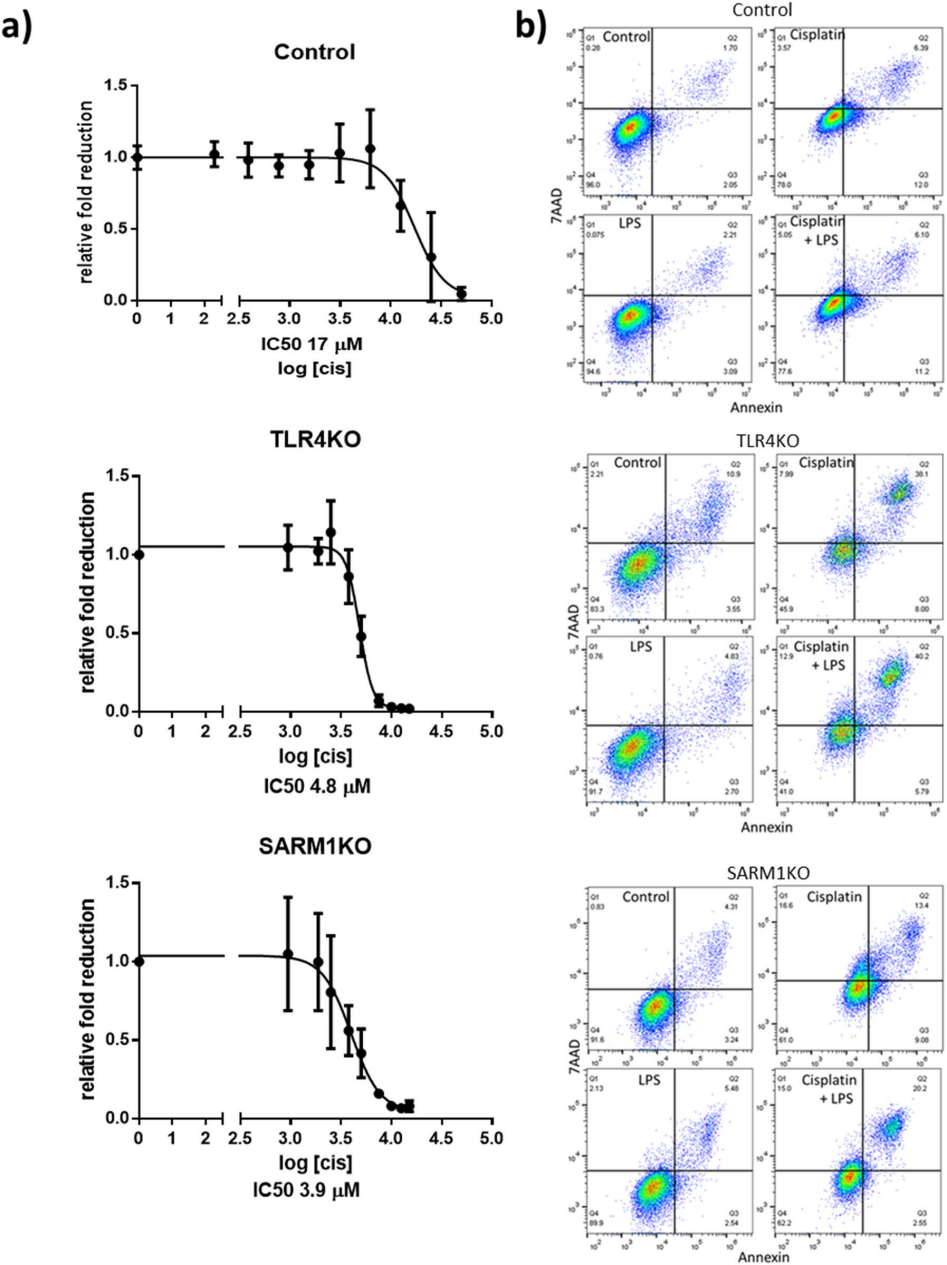
TLR4KO and SARM1KO cells are more sensitive to chemotherapy than parental HeLa cells. (**a**) Determination of half maximal inhibitory concentration (IC50) of cisplatin for TLR4KO, SARM1KO and parental HeLa cells. Cell viability was measured after 72 h of cisplatin treatment using PrestoBlue reagent assay. (**b**) Representative flow cytometry plots of apoptosis induced in parental unedited (top), TLR4KO (middle) and SARM1KO (bottom) cells after treatment with 4.7 µM cisplatin and/or 10 µg/mL LPS for 72 h, using 7-AAD and Annexin markers. Data in A displayed as mean ± SD.

To determine if TLR4 and SARM1 impact the response of cervical cancer cells to chemotherapy, we treated parental, TLR4KO and SARM1KO cells with different concentrations of cisplatin. Figure 3a shows that TLR4KO and SARM1KO cells had higher sensitivity to cisplatin than parental HeLa cells. This enhanced effect is also illustrated by the calculation of the IC50 (concentration at which we observe a 50% reduction in cellular viability): around 4.8 µM for TLR4KO, 3.9 µM for SARM1KO, and 17 µM for parental cells.

### TLR4KO induces apoptosis after cisplatin treatment

**Figure 4:**
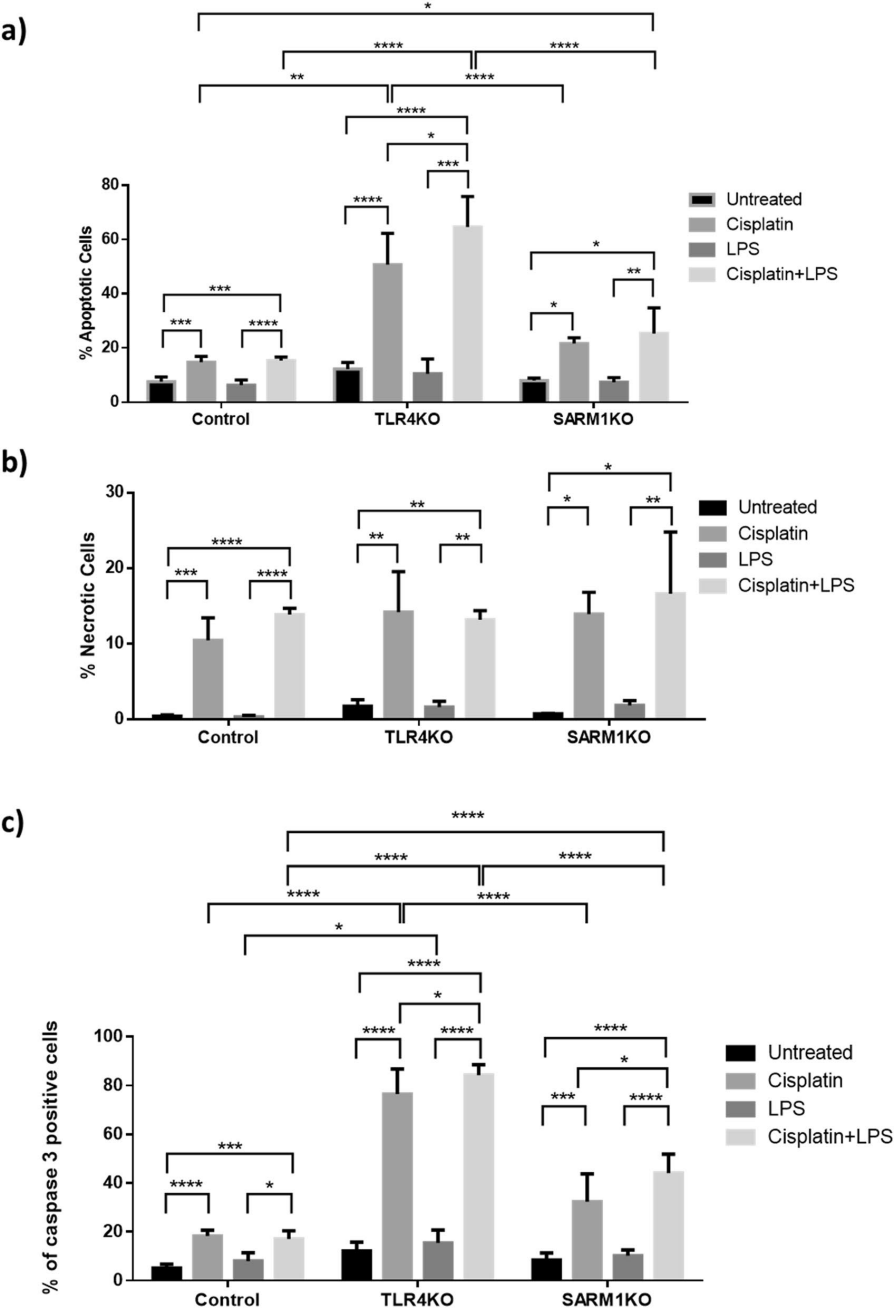
Apoptosis induced by cisplatin is differentially affected by TLR4 or SARM1 knockout. (**a**) Percentage of apoptotic cells determined by Annexin staining and flow cytometry analysis in TLR4KO, SARM1KO and parental HeLa cells after 4.7 µM cisplatin and/or 10 µg/mL LPS treatment for 72 h. (**b**) Percentage of necrotic cells determined by 7-AAD staining and flow cytometry analysis in TLR4KO, SARM1KO and parental cells after 4.7 µM cisplatin and/or 10 µg/mL LPS treatment for 72 h. (**c**) Percentage of cells with activated caspase 3 by flow cytometry analysis in TLR4KO, SARM1KO and parental cells after 4.7 µM cisplatin and/or 10 µg/mL LPS treatment for 72 h. Data displayed as mean ± SD (*p ≤ 0.05; **p ≤ 0.01; ***p ≤ 0.001; ****p ≤ 0.0001).

We then investigated whether knocking out either *TLR4* or *SARM1* induces cell death. In the case of TLR4KO cells, we observed that cisplatin treatment significantly increased apoptosis when compared to parental HeLa cells, and this effect was enhanced when cisplatin was combined with LPS (Figs. 3b and 4a). Interestingly, more SARM1KO cells went through apoptosis only when treated with both cisplatin and LPS, as compared to parental cells treated with cisplatin alone. We observed no differences in the induction of necrosis between cells (KO or parental), or between LPS or cisplatin treatments conditions. Nevertheless, parental and KO cells responded to cisplatin with and without LPS, showing a significant increase in necrosis rate, despite individual differences in susceptibility (Fig. 4b).

To confirm that apoptosis was being triggered, we measured the amount of activated caspase 3 by flow cytometry. Although cisplatin induced caspase 3 activation in both parental and KO cells, the effect was more pronounced in TLR4KO cells compared to unedited HeLa cells (Fig. 4c and Supplementary Fig. S3). Even though SARM1KO cells also showed higher levels of activated caspase 3 after cisplatin treatment, this increase was not significantly higher than the levels observed in parental cells (Fig. 4c and Supplementary Fig. S3). In addition, when using cisplatin combined with LPS, both TLR4KO and SARM1KO cells showed an increase in active caspase 3 levels compared to parental cells (Fig. 4c and Supplementary Fig. S3). LPS alone increased apoptosis levels in TLR4KO cells compared to parental cells (Fig. 4c). Taken together, our data shows that TLR4 depletion sensitizes HeLa cells to chemotherapy-induced apoptosis independent of LPS exposure whereas deleting *SARM1* sensitizes cells to chemotherapy only in the presence of LPS.

### TLR4 and SARM1 modulate ROS production

**Figure 5:**
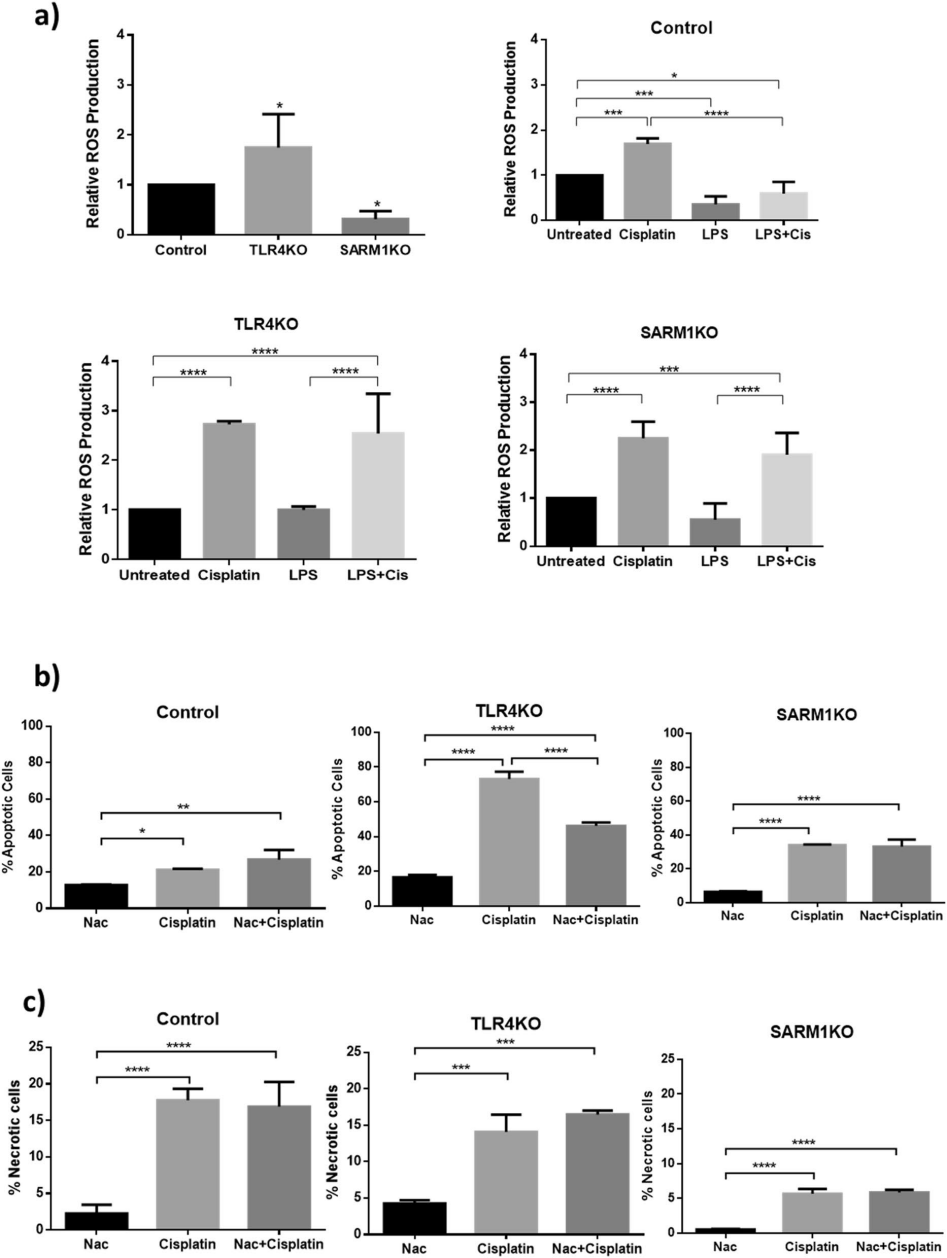
N acetyl L cysteine (NAC) reduces apoptosis in TLR4KO cells after cisplatin treatment. (**a**) ROS levels after cisplatin and LPS treatment in TLR4KO, SARM1KO and parental cells measured by flow cytometry. (**b**) Percentage of apoptotic cells determined by Annexin staining and flow cytometry analysis after 4.7 µM of cisplatin and 100 µM of NAC treatment for 72 h in parental, TLR4KO and SARM1KO cells. (**c**) Percentage of necrotic cells determined by 7-AAD and flow cytometry analysis after 4.7 µM of cisplatin and 100 µM NAC treatment for 72 h in TLR4KO, SARM1KO and parental cells. Data displayed as mean ± SD. (*p ≤ 0.05; **p ≤ 0.01; ***p ≤ 0.001; ****p ≤ 0.0001).

Cisplatin and the TLR pathway modulate production of reactive oxygen species (ROS), which induces cell damage and stimulates apoptosis. Therefore, we measured ROS production in parental, TLR4KO and SARM1KO HeLa cells exposed to the same treatments performed before (Fig. 5a). We noticed that, compared to parental HeLa cells, basal ROS levels were lower in SARM1KO and higher in TLR4KO. Cisplatin increased ROS levels in edited and unedited cells, whereas LPS lowered ROS levels only in unedited HeLa cells and had no significant effect on ROS levels in TLR4KO and SARM1KO cells. When cells were treated with cisplatin and LPS, both TLR4KO and SARM1KO cells showed an increase in ROS levels similar to that obtained when cells were treated with cisplatin alone. Both TLR4KO and SARM1KO cells modulated ROS levels although LPS decreased ROS production only in parental HeLa cells.

Since cisplatin can induce apoptosis through ROS production, we exposed cells to a ROS inhibitor, N acetyl L cysteine (NAC) to determine if the chemotherapy sensitivity of knockouts were related to ROS levels (Supplementary Fig. S4). We observed that NAC did not alter cisplatin-induced apoptosis of SARM1KO or parental cells. On the other hand, apoptosis levels were significantly lower in NAC-treated TLR4KO cells exposed to cisplatin (Fig. 5b). No effect in necrosis was observed (Fig. 5c). Therefore, our results suggest that the apoptosis observed in TLR4KO cells in response to cisplatin is related to an increase of cellular ROS levels.

### Expression of TLR4 and SARM1 alter expression of genes involved in tumorigenic and inflammatory processes

**Figure 6:**
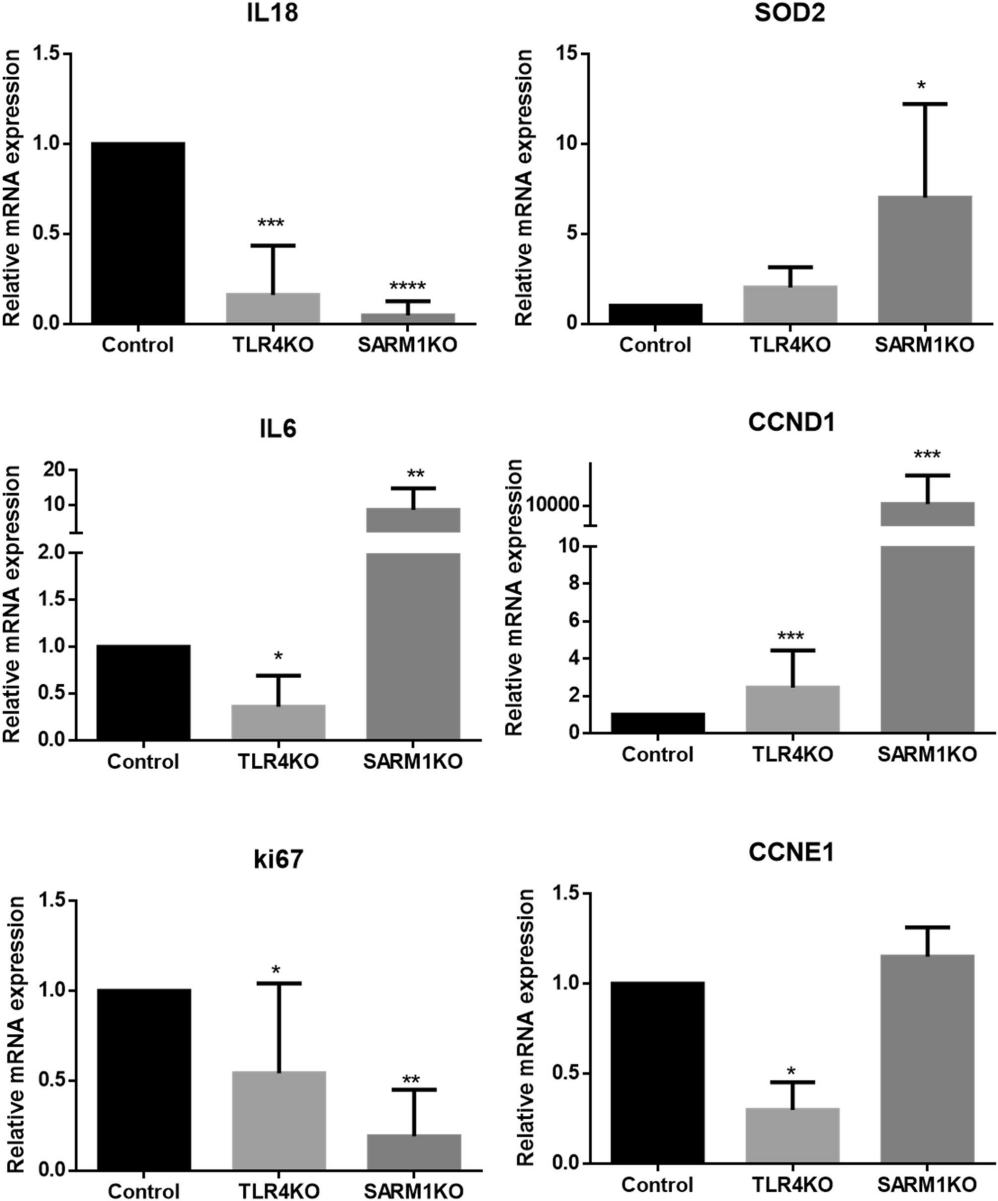
TLR4KO and SARM1KO modulate the expression of genes related to inflammation and proliferation. Data obtained by quantitative RT-PCR analysis of three biological replicates for *IL18*, *IL6*, *CCND1*, *CCNE1*, *SOD2* and *Ki-67* in TLR4KO, SARM1KO and parental HeLa cells. Data displayed as mean ± SD. (*p ≤ 0.05; **p ≤ 0.01; ***p ≤ 0.001; ****p ≤ 0.0001).

To better understand the effect of knocking out *TLR4* and *SARM1*, we evaluated the expression profile of genes potentially modulated by TLR and related to several steps in the tumorigenic and inflammatory processes. We checked the expression levels of *IL6* and *IL18* (which are related to TLR triggering and inflammation), Superoxide Dismutase 2 (*SOD2*, responsible for ROS and oxidative stress modulation), Cyclins D1 and E1 (*CCND1* and *CCNE1*, respectively, which are involved in cell cycle progression), and *Ki-67* (a marker of cell proliferation) in HeLa cells depleted of TLR4 or SARM1, in comparison to the parental HeLa cells (Fig. 6).

In both TLR4KO and SARM1KO cells, we observed lower levels of *IL18* compared to parental cells. On the other hand, while TLR4KO cells showed decreased levels of *IL6*, we observed increased levels of *IL6* in SARM1KO cells, as compared to the unedited HeLa cells. *SOD2* expression was higher in SARM1KO cells, but not TLR4KO cells, compared to parental cells. *CCND1* expression levels increased in both SARM1KO and TLR4KO cells, while *CCNE1* levels decreased significantly only in TLR4KO cells. In addition, *KI-67* expression levels were lower in TLR4KO and SARM1KO cells compared to parental HeLa cells (Fig. 6).

In conclusion, lack of TLR4 and SARM1 in HeLa cells changed the expression of genes related to inflammatory response, cell cycle and proliferation.

## Discussion

TLR4 has been implicated in both triggering innate immune response against bacteria and colon cancer development^26^. In addition, in ovarian tumors, its activation leads to increased cell proliferation and cytokines and chemokines production^27^. Nevertheless, TLR4 has different roles depending on tumor type. In hepatocellular carcinoma, for example, its absence leads to more DNA damage, inhibition of senescence and tumor progression^28^. In pancreatic tumors, LPS stimulates tumor development and increases invasiveness in vivo through activation of NF-κB via TLR4 signaling^29^. LPS treatment has also been implicated in the establishment of cervical tumors in a mouse model, while *TLR4* silencing led to the formation of smaller tumors^30^.

Several studies also showed a role for TLR4 in tumorigenesis and therapy resistance. There is no consensus of a single set of genes involved in these processes, but some studies suggest an association between TLR4 and epithelial-to-mesenchymal transition through STAT3 activation^31^ as well as a role in proliferation through the activation of pathways such as MAPK^32^. Studies of chemotherapy resistance related to TLR4 activation also revealed changes in the expression of genes related to apoptosis and cell cycle^33^.

Few studies have shown an association between increased TLR4 expression in HPV-positive cervical tumor samples and resistance to apoptosis^15,34,35^. We have previously reported that TLR4 had higher expression levels in HPV-positive tumor cells and in normal cells expressing HPV16 E6 and E7 oncoproteins^15^. After further investigation, our results confirmed the role of TLR4 in cell survival, proliferation and response to cisplatin in HeLa cells. Lack of TLR4 in this cell line led to increased ROS production and consequently increased apoptosis after treatment.

Another poorly understood protein related to TLR4 is SARM1, a negative regulator of TLR signaling^36^. Other functions have been described for SARM1 in neuronal cell models, such as modulating mitophagy^37^, increasing resistance to TNF treatment-induced apoptosis^38^, and triggering sarmoptosis, a recently uncovered form of programmed cell death, after ROS production^39^.

However, no relationship between SARM1 and cancer or HPV infection has been reported until now. This is the first study to suggest its possible role in HPV tumorigenesis. Initially, we described the increased expression of its gene in cervical tumor cell lines^15^; here we are reporting its role in cell survival and response to chemotherapy.

Also, we describe changes in the expression of a set of genes after knockout of *SARM1* or *TLR4*. One of the genes is *SOD2*, a known regulator of oxidative stress and ROS levels. SOD2 has been previously associated with cervical cancer and high grade lesions of the cervix^40^. In addition, higher *SOD2* expression is associated with presence of lymph node metastasis in penile cancer^41^. SOD2 is involved in resistance to TNF treatment in keratinocytes^42^ and a relationship between LPS treatment and SOD2 expression and activity has also been demonstrated^43^. Additionally, we are the first group to report *SOD2* and ROS regulation by *SARM1* expression.

TLR pathway is also involved in interleukin expression. We analyzed the expression of *IL18* and *IL6*, interleukins linked to inflammation and tumorigenesis^44^. High risk HPV promote upregulation of IL6 and TLR4, resulting in an inflammatory environment that can contribute to tumor development^45,46^. IL6 can also lead to STAT3 activation, and this pathway contributes to cell proliferation and survival of cervical cancer cells^47^. Here we show that the expression of *IL6* and *IL18* is modulated by TLR4 and SARM1 in cervical cancer cells.

We also observed a decrease in proliferation after knocking out *SARM1* or *TLR4*, which prompted us to check *CCND1*, *CCNE1* and *Ki67*. The proliferation marker Ki-67 is associated with high risk HPV and lesion progression^48^, and is widely used as a biomarker, together with p16, to identify HPV infected tissues^49^. Cyclins D1 and E1 (encoded by *CCND1* and *CCNE1*, respectively) are responsible for cell cycle progression. The oncoprotein E6 of HPV can activate the transcription factor eIF4E, and its inhibition in cervical cancer cell lines leads to *CCND1* downregulation^50^. Interestingly, cyclin D1 leads to the release of E2F by inducing phosphorylation of pRB, a protein that is already targeted by HPV oncoprotein E7^51,52^. Despite the role of *CCNE1* in the proliferation of normal cell lines, it has been reported as a biomarker for chemotherapy resistance in ovarian cancer and for prognosis in breast cancer^53,54^. Both *CCND1* and *CCNE1* are upregulated in squamous cervical carcinomas^55^. Our data showed modulation of all three genes, *Ki67, CCND1* and *CCNE1*, by TLR4 and SARM1.

In conclusion, we demonstrate here that, in HeLa cells, knock out of *SARM1* drastically reduced cell viability and proliferation while lack of TLR4 significantly increased apoptosis and ROS production after cisplatin treatment. Further studies are warranted to explore the potential use of these findings in the clinic.

## Methods

### Plasmids for gene editing and cell line

The following plasmids were used for CRISPR/Cas9 editing: pX330A^23^, pX330S^23^ and PX459 (pSpCas9(BB)-2A-Puro)^24^ (# 58766, # 58778, and # 62988, Addgene, Watertown, MA).

The pX330A and pX330S vectors were used in combination to build a multiplex system for *TLR4* editing, while the pX459 vector was used alone for *SARM1* editing as previously described^24^.

Guide sequences for gene editing were designed using a specific software (crispor.tefor.net): SARM1 G # F–CACCGCGCCTGCTTCAGCGCGGACA; SARM1 G # R–AAACTGTCCGCGCTGAAGCAGGCGC; TLR4 G # 1F–CACCGATGCCCCATCTTCAATTGTC; TLR4 G # 1R–AAACGACAATTGAAGATGGGGCATC; TLR4 G # 2F–CACCGGGGTACAGGATGCAATTGGC; TLR4 G # 2R–AAACGCCAATTGCATCCTGTACCCC.

HeLa cells were purchased from American Type Culture Collection (ATCC® CCL-2™, Lot Number: 59681574) in December 2012. ATCC certified cells through several tests, including: mycoplasma contamination, COI assay, STR analysis, Sterility and Human pathogenic virus presence. Upon receipt HeLa cells were divided, frozen and kept in low passage for all subsequent experiments. Cells were grown in MEM culture medium supplemented with 10% fetal bovine serum (M10 medium) at 37ºC with 5% CO_2_ and transfected using the FuGENE HD reagent (Promega, Madison, WI) according to the manufacturer's protocol at a 3:1 ratio between reagent and plasmids, followed by selection with 2 µg/ml of puromycin. Individual clones were isolated by serial dilution of selected cells in 96-well plates. Alterations were confirmed by Sanger sequencing (Supplementary Fig. S1). Knockout cells were obtained before passage 10.

### Western Blot

To evaluate whether the genes of interest were knocked out, protein extracts were analyzed by western blotting using polyclonal antibodies specific for SARM1 (ab115561, Abcam, Cambridge, UK) and TLR4 (ab13556, Abcam, Cambridge, UK). Cells were lysed with a buffer containing 150 mM NaCl, 5 mM EDTA, 50 mM Tris-HCI pH 8.0, 1% NP-40, 0.5% Sodium deoxycholate and 0.1% SDS supplemented with protease inhibitors (Sigma-Aldrich, St. Louis, MO). Protein concentration was determined using Bio-Rad protein assay (Bio-Rad, Hercules, CA).

### Cell viability assay

Cell viability analysis was performed using the PrestoBlue ™ Cell Viability Reagent kit (ThermoFisher Scientific, Waltham, MA). Cells were seeded in 96-well plates, and 100 μl of complete medium plus 10 μl of PrestoBlue reagent was added to each well. The plates were incubated for 1 h at 37 °C protected from light. Absorbance was measured at 570 and 600 nm. For viability analysis, cells were previously treated with Lipopolysaccharide (LPS) (InvivoGen, San Diego, CA) at 10 µg/mL with and without cisplatin at 4.7 µM (Accord Farmacêutica, São Paulo, Brazil) and analyzed after 72 h of treatment.

### Clonogenic, proliferation and migration assays

For clonogenic assays, cells were seeded at low density (1000 cells per well) in 6-well plates. After 2 weeks, cells were fixed with formalin and stained with crystal violet (0.05% w/v). The number of colonies formed was counted automatically using the ImageJ software (National Institutes of Health, Bethesda, MD). For proliferation assays, 5000 cells were seeded per well in 24-well plates and counted every day in triplicates. For migration assays, 3 × 10^6^ cells were seeded in 6-well plates; 24 h later, cells were treated with 10 µg/ml of mitomycin C (Sigma-Aldrich, St. Louis, MO) for 2 h at 37ºC. A wound healing assay was performed to observe cell migration, after the mitomycin C treatment. Photos were taken at 0 h, 24 h and 48 h, and the area not occupied by cells was measured using the ImageJ software.

### Cell death assays and ROS production measurement

Apoptosis was determined using the CellEvent™ Caspase-3/7 Green Detection Reagent kit (ThermoFisher Scientific, Waltham, MA) following the manufacturer's protocol. Apoptosis and necrosis were also determined using double staining with the eBioscience™ 7-AAD Viability Staining Solution and eBioscience™ Annexin V-FITC Apoptosis Detection Kit (ThermoFisher Scientific, Waltham, MA) and analyzed by flow cytometry.

For reactive oxygen species (ROS) determination, cells were cultured in 6-well plates with 10 µg/mL of LPS, 4.7 µM of cisplatin and 100 µM N-acetyl-l-cysteine (NAC) for 72 h, followed by 30 min of incubation with the DCFDA/H2DCFDA reagent (Cellular ROS Assay Kit, ThermoFisher Scientific, Waltham, MA) and analysis by flow cytometry.

For cell cycle analysis, 10^5^ cells were seeded per well in 6-well plates and, after 24 h, cells were treated with 10 µg/ml mitomycin for 2 h. Cells were fixed with 70% ethanol, washed with PBS and treated with 5 µl of RNAse (10 mg/ml, ThermoFisher Scientific, Waltham, MA), 0.5 µl of 7-AAD and 0.5 µl of Triton X-100 (Sigma-Aldrich, St. Louis, MO) diluted in 500 µl of PBS for 30 min at 37 °C. Cells were then analyzed by flow cytometry.

All experiments were performed on a BD Accuri™ C6 cytometer (BD Biosciences, CA) and the results analyzed with FlowJo software (BD, Franklin Lakes, NJ, version V10).

### Quantitative polymerase chain reaction (qPCR)

RNA was extracted with TRIzol as described by the manufacturer (Thermo-Fisher, Waltham, MA). After DNase treatment (Promega, Madison, WI), 1 µg of RNA was used for cDNA synthesis and qPCR with GoTaq® 2-Step RT-qPCR System (Promega, Madison, WI) following the manufacturer’s protocol. Primers used were: IL18F TGGCTGTAACTATCTCTGTGAA; IL18R TGTCCTGGGACACTTCTCTG; SOD2 F TGAACAACCTGAACGTCACC; SOD2RGTAGTAAGCGTGCTCCCACA; IL6 F CCACACAGACAGCCACTCAC; IL6 R AGGTTGTTTTCTGCCAGTGC; CCND1 F TGAAGGAGACCATCCCCCTG; CCND1 R TGTTCAATGAAATCGTGCGG; CCNE1 F AAATGGCCAAAATCGACAGG; CCNE1 R CGAGGCTTGCACGTTGAGTT; MKI67 F TGTGCCTGCTCGACCCTACA; MKI67 R TGAAATAGCGATGTGACATGTGCT. Expression analysis was performed using the ΔΔCt method^25^.

### Statistical analysis

All experiments were performed at least in triplicates. Results were analyzed using two-way ANOVA with Prism software (Graphpad, version 6, San Diego, CA).

## Supplementary Information

Supplementary Information.
