# Supplementary material for: TLR4 and SARM1 modulate survival and chemoresistance in an HPV-positive cervical cancer cell line
Source: Sci Rep. 2022 Apr 25;12:6714. doi: 10.1038/s41598-022-09980-6 (PMC9039070; doi:10.1038/s41598-022-09980-6)
Supplement: Supplementary file 1 — Supplementary Information. [file 41598_2022_9980_MOESM1_ESM.pdf]

SARM1 Original

MVLTLLLSAY KLCRFFAMSG PRPGAERLAV PGPDGGGGTG PWWAAGGRGP
REVSPGAGTE VQDALERALP ELQQALSALK QAGGARAVGA GLAEVFQLVE
EAWLLPAVGR EVAQGLCDAI RLDGGLDLLL RLLQAPELET RVQAARLLEQ
ILVAENRDRV ARIGLGVILN LAKEREPVEL ARSVAGILEH MFKHSEETCQ
RLVAAGGLDA VLYWCRRTDP ALLRHCALAL GNCALHGGQA VQRRMVEKRA
AEWLFPLAFS KEDELLRLHA CLAVAVLATN KEVEREVERS GTLALVEPLV
ASLDPGRFAR CLVDASDTSQ GRGPDDLQRL VPLLDSNRLE AQCIGAFYLC
AEAAIKSLQG KTKVFSDIGA IQSLKRLVSY STNGTKSALA KRALRLLGEE
VPRPILPSVP SWKEAEVQTW LQQIGFSKYC ESFREQQVDG DLLLRLTEEE
LQTDLGMKSG ITRKRFFREL TELKTFANYS TCDRSNLADW LGSLDPRFRQ
YTYGLVSCGL DRSLLHRVSE QQLLEDCGIH LGVHRARILT AAREMLHSPL
PCTGGKPSGD TPDVFISYRR NSGSQLASLL KVHLQLHGFS VFIDVEKLEA
GKFEDKLIQS VMGARNFVLV LSPGALDKCM QDHDCKDWVH KEIVTALSCG
KNIVPIIDGF EWPEPQVLPE DMQAVLTFNG IKWSHEYQEA TIEKIIRFLQ
GRSSRDSSAG SDTSLEGAAP MGPT-

SARM1KO

MVLTLLLSAY KLCRFFAMSG PRPGAERLAV PGPDGGGGTG PWWAAGGRGP
REVSPGAGTE VQDALERALP **RSSNWWRRPG CCRPWAAR**.

TLR4 Original
1......720 NIIHEGFHKS RKVIVVVSQH FIQSRWCIFE YEIAQTWQFL SSRAGIIFIV
LQKVEKTLLR QQVELYRLLS RNTYLEWEDS VLGRHIFWRR LRKALLDGKS WNPEGTVGTG
CN**WQEATSI**

TLR4KO Allele 1
1......720 NIIHEGFHKS RKVIVVVSQH FIQSRWCIFE YEIAQTWQFL SSRAGIIFIV
LQKVEKTLLR QQVELYRLLS RNTYLEWEDS VLGRHIFWRR LRKALLDGKS WNPEGTVGTG
CN**LSEEEK**

TLR4KO Allele 2
1......720 NIIHEGFHKS RKVIVVVSQH FIQSRWCIFE YEIAQTWQFL SSRAGIIFIV
LQKVEKTLLR QQVELYRLLS RNTYLEWEDS VLGRHIFWRR LRKALLDGKS WNPEGTVGTG
CN.

Supplementary Figure 1: SARM1KO and TLR4KO predicted protein sequence alterations after introduction of mutations by CRISPR/Cas9, based on Sanger DNA sequencing data.

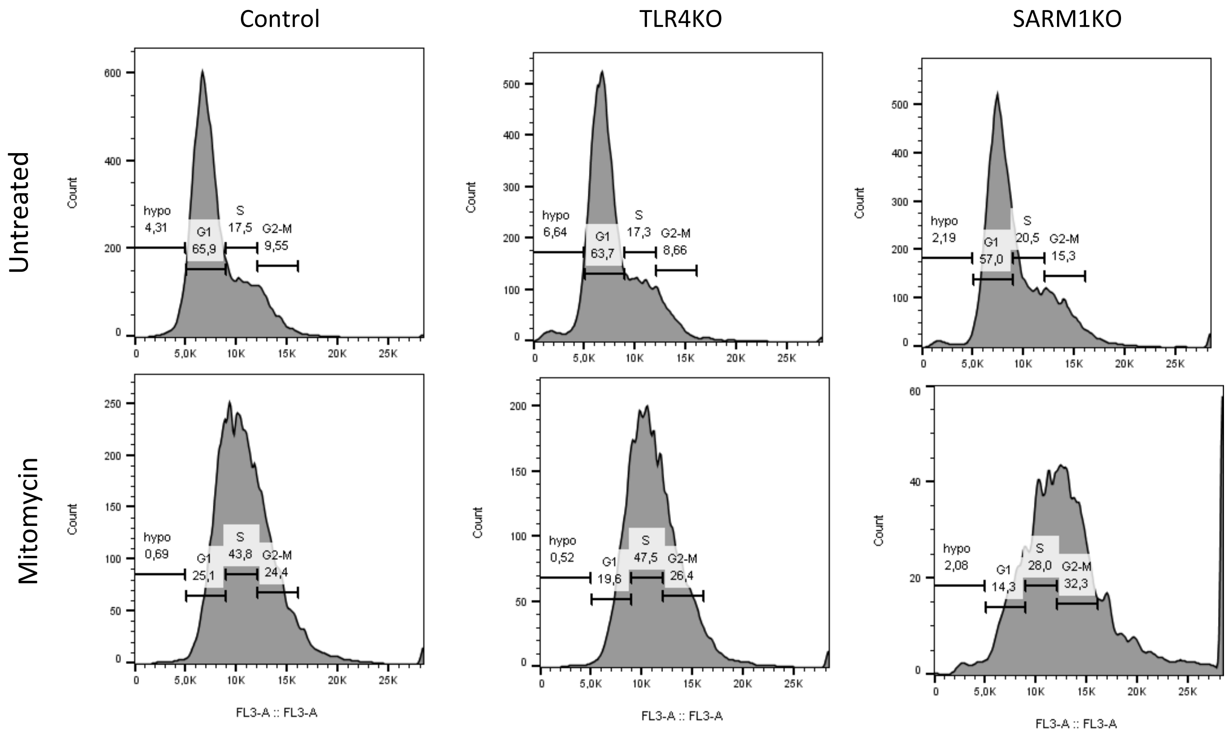

Supplementary Figure 2: S phase arrest after 10 µg/ml mitomycin treatment for 2 hours. Flow cytometry analysis in TLR4KO, SARM1KO and parental cells.

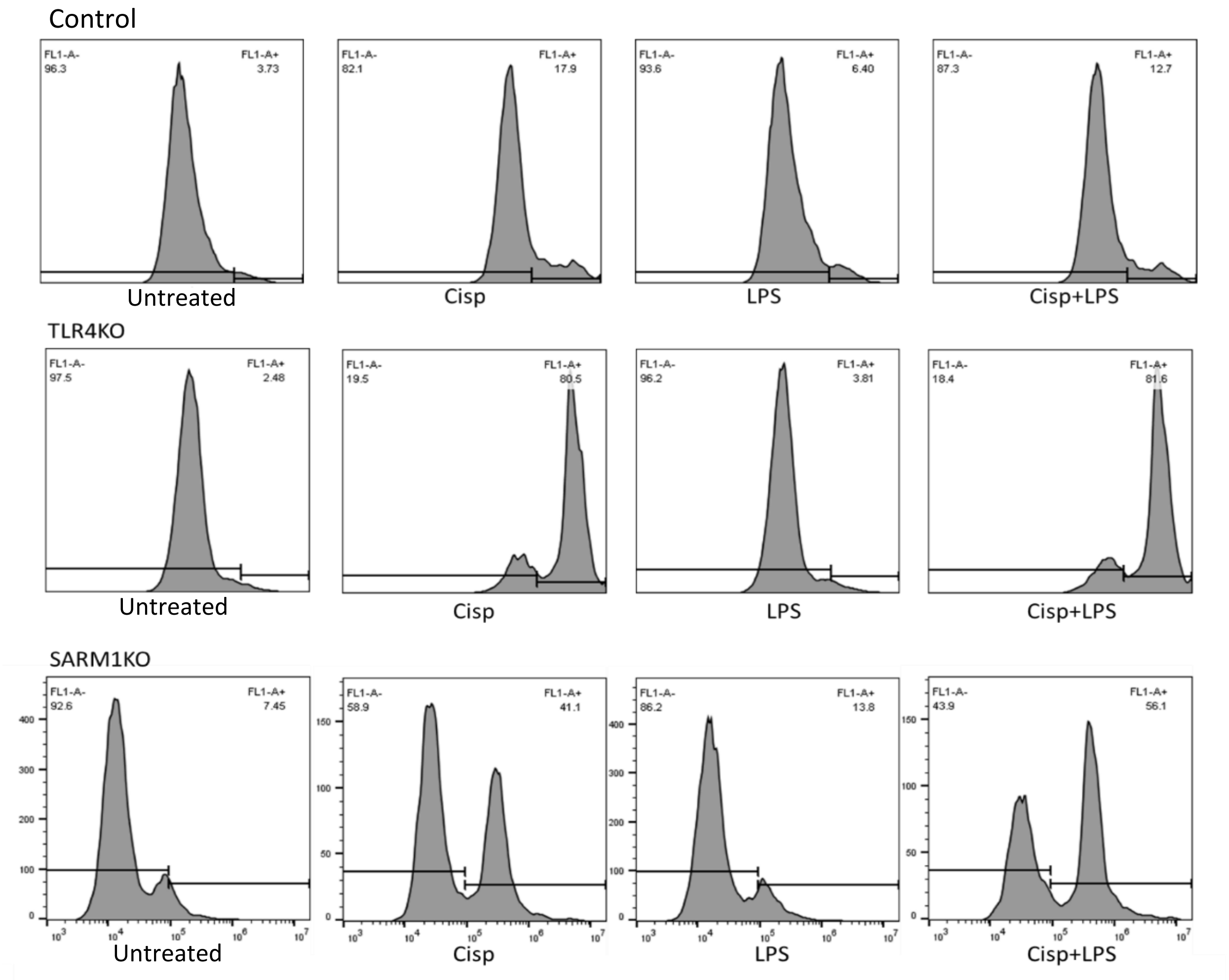

Supplementary Figure 3: Representative flow cytometry histograms of activated caspase 3 levels in parental (top), TLR4KO (middle) and SARM1KO (bottom) cells after 4.7 µM cisplatin and/or 10 µg/mL LPS treatments for 72 hours.

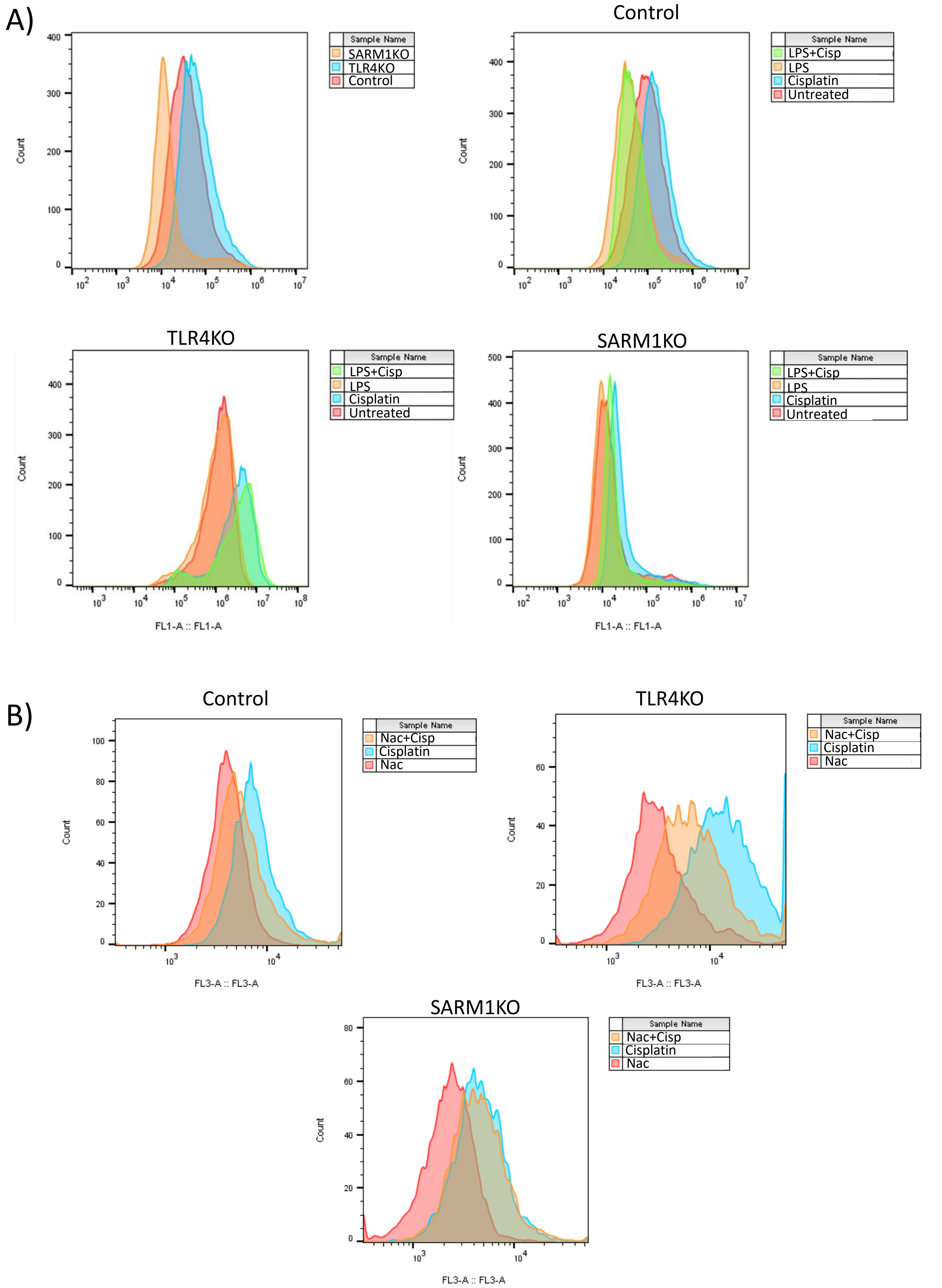

Supplementary Figure 4: Representative flow cytometry histograms of ROS levels in TLR4KO, SARM1KO and parental cells. A) After 4.7 µM of cisplatin treatment for 72 hours. B) After 4.7 µM of cisplatin and 100 µM of NAC treatment for 72 hours, in combination with cisplatin.

Uncropped Western Blots

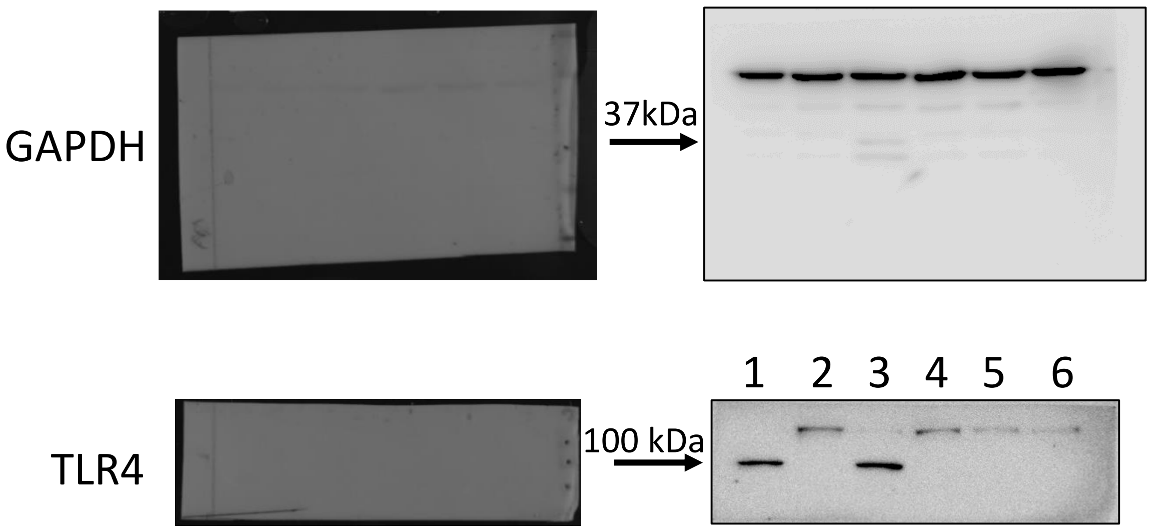

1 HeLa Control
2 HeLa TLR4 KO clone 1
3 HeLa TLR4 KO clone 2
4 HeLa TLR4 KO clone 3
5 HeLa TLR4 KO clone 4
6 HeLa TLR4 KO clone 5

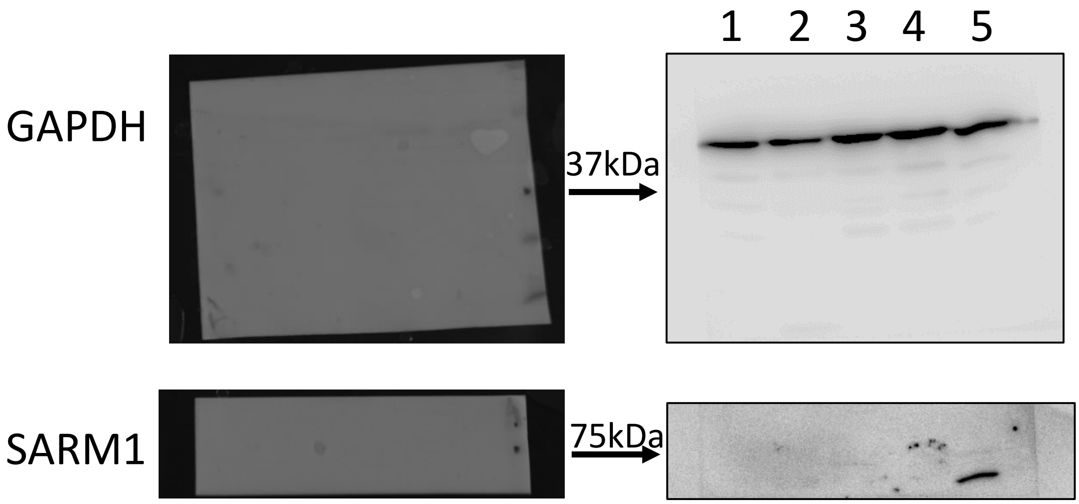

1 HeLa SARM1 KO clone 1
2 HeLa SARM1 KO clone 2
3 HeLa SARM1 KO clone 3
4 HeLa SARM1 KO clone 4
5 HeLa Control
